# Anatomical Possibilities of the Alveolar Bone at the Upper Second Premolar Level

**DOI:** 10.3390/medicina60050726

**Published:** 2024-04-27

**Authors:** Alexandru Nicolae Mureşan, Carol Antonio Dandoczi, Răzvan Costin Tudose, Sorin Hostiuc, Mugurel Constantin Rusu

**Affiliations:** 1 Division of Anatomy, Department 1, Faculty of Dentistry, “Carol Davila” University of Medicine and Pharmacy, RO-020021 Bucharest, Romaniarazvan-costin.tudose0721@stud.umfcd.ro (R.C.T.);; 2 Research Department, “Dr. Carol Davila” Central Military Emergency Hospital, RO-010825 Bucharest, Romania; 3 Division of Legal Medicine and Bioethics, Faculty of Dentistry, “Carol Davila” University of Medicine and Pharmacy, RO-020021 Bucharest, Romania

**Keywords:** maxilla, maxillary sinus, nasal fossa, alveolar bone

## Abstract

*Background and Objectives*: The upper posterior teeth are typically regarded as being exclusively inferior to the maxillary sinus (MS). The expansion of the nasal fossa above the maxillary alveolar base (MAB) needs better investigation. The hypothesis was raised that the MAB in the upper premolar region, which is usually addressed by surgeons for the elevation of the antral floor, is not exclusively beneath the MS. Therefore, we aimed to document the possible upper relations of the MAB as antral, nasal, or both. *Materials and Methods*: A total of 145 CBCT scans were used to study four types of MAB: type 1—antral; type 2—antral with a palatal recess; type 3—antral and nasal; type 4—nasal. In type 2, the orthoradial width of the alveolar bone, the rectilinear width of the antral floor, and the maximum depth of the palatal recess were measured. For type 3, the MAB width and the straight widths of the antral and nasal segments of the MAB were measured. *Results*: Type 1 was found in 67.24%, type 2 in 13.45%, type 3 in 16.21%, and type 4 in 3.1% of the 290 MSs investigated. Palatal recesses were found in 11.72% of the MSs on the right side and 15.17% of the MSs on the left side. Types 1 and 2 exhibited strongly statistically significant bilateral symmetry (Pearson’s Chi2 = 86.42, *p* < 0.001). Type 3 correlated equally with contralateral types 1 and 3. The bilateral symmetry for types 1–3 was stronger in the males (Pearson’s Chi2 = 47.83, *p* < 0.001) than in the females (Pearson’s Chi2 = 56.96, *p* < 0.001). There were no statistically significant associations between sex and the unilateral anatomical type. *Conclusions*: The MAB in the upper second premolar area should not be considered to be exclusively antral during surgeries or in anatomical teaching.

## 1. Introduction

The floor of the maxillary sinus (MS) is encased and is formed from the lower third of the medial wall and the bucco-alveolar wall of the MS. The antral floor is typically made by the alveolar process of the maxilla [1]. Studies of the antral floor have focused on specific dental–antral relationships, assuming the hypothesis of a unique possibility of the localisation of the MS being exclusively superior to the alveolar bone [1,2,3,4]. The anatomy of the antral floor is of the utmost importance for augmentation procedures during endosseous implant placement [5,6]. Insufficient bone quantity sometimes causes the implant to migrate to the MS [7].

A previous report of MS hypoplasia detailed that the lateral nasal wall protruded within the respective maxillary bone to reach above the vestibular cortical plate of the alveolar process, and that expansion was limited to the premolar and first molar regions [8]. As such, the expansion of the nasal fossa above the maxillary alveolar bone is an anatomic possibility, the incidence of which needs to be established. Three-dimensional computed tomography technologies have greatly improved our ability to explore and estimate the limits of the MS and nasal fossa (NF) [9]. The MAB of the postmaxilla is clinically relevant in planning dental implantations, endodontic procedures, and orthodontic treatment [10]. Dental surgeons can benefit from studying the antral and nasal floor by Cone Beam Computed Tomography (CBCT) when using different approaches for the elevation of the sinus floor for placing dental implants to assist in reconstructions [11]. CBCT scans are the gold standard for dental implant planning worldwide [12].

The hypothesis was raised that the MAB in the upper premolar region, which is usually addressed by surgeons for the elevation of the antral floor, is not exclusively beneath the MS. We therefore aimed to document the possible upper relations of the MAB as antral, nasal, or both.

## 2. Materials and Methods

An archived batch of CBCT scans of 150 cases was used. Inclusion criteria for the study were high-quality CBCT scans with no distortions or added artefacts, a complete vertical scan path of the maxilla and the presence of adjacent or opposing teeth to the edentulous span so that the location of the edentulous ridges corresponding to the tooth site could be identified. Exclusion criteria were incomplete or unclear scans, distorted anatomical structures or other added artefactual images and the impossibility of location of the second upper premolar area in edentulous cases. There were 62 male cases; 2 cases were excluded, and 60 were included in the study. There were 88 female cases; 3 cases were excluded, and 85 were included in the study. Thus, we retrospectively followed the targeted anatomical variables in 145 cases.

**Figure 1:**
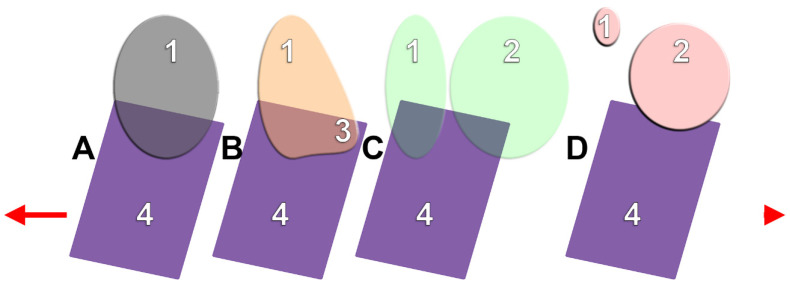
Topographic patterns of the maxillary alveolar base (MAB). Bucco-palatal sections. (**A**): type 1; (**B**): type 2; (**C**): type 3; (**D**): type 4. 1. Maxillary sinus; 2. nasal fossa; 3. palatal recess of maxillary sinus; 4. MAB. Buccal (arrow) and palatal (arrowhead) orientation.

Four possible topographic types (Figure 1) of the MAB were defined: type 1: exclusively antral MAB; type 2: antral MAB with a palatal recess of the antral floor interposed between the MAB and the NF; type 3: antral and nasal MAB, participating directly in both the antral and nasal floors; and type 4: exclusively nasal MAB.

On the orthoradial sections at the upper second premolar level, we measured the type 2 MABs on each side, the orthoradial width of the alveolar bone (process), the rectilinear width of the antral floor, and the maximum depth of the palatal recess of the MS. For the type 3 MABs, the following were measured: (a) the MAB width, considered as either the buccopalatal thickness of the alveolar process in dentate or that of the alveolar bone in edentulous individuals, in the middle 1/3; (b) the straight width of the antral segment of the MAB; (c) the straight width of the nasal segment of the MAB. We excluded types 1 and 4 from measurements because only in types 2 and 3 is the palatal segment of the MAB related to a pneumatic space; which is either a palatal recess of the MS in type 2 or the NF in type 3.

We recorded the following variables (vertical topography) for types 1-3: ‘a’: the antral floor higher than the nasal floor; ‘b’: the antral floor flush with the nasal floor; ‘c’: the antral floor lower than the nasal floor.

We classified the palatal recesses of the antral floor as open (O) when they communicated widely with the antral cavity, and closed (C) when almost wholly separated from the main antral cavity. 

We further used a series of statistical tests to assess the results. To determine the number of cases in each variant, we used frequencies and gender distribution. To assess significant associations between qualitative variables, we used the Pearson Chi2 test. We used ANOVA tests to identify whether there were differences in the morphometric determinations. We applied the Pearson correlation test to assess bilateral associations between the measured variables. We used IBM SPSS Statistics for Mac os Version 28.0. (IBM Corp, Armonk, NY, USA) to perform statistical analyses.

## 3. Results

### 3.1. Types of the Antral Floor

**Figure 2:**
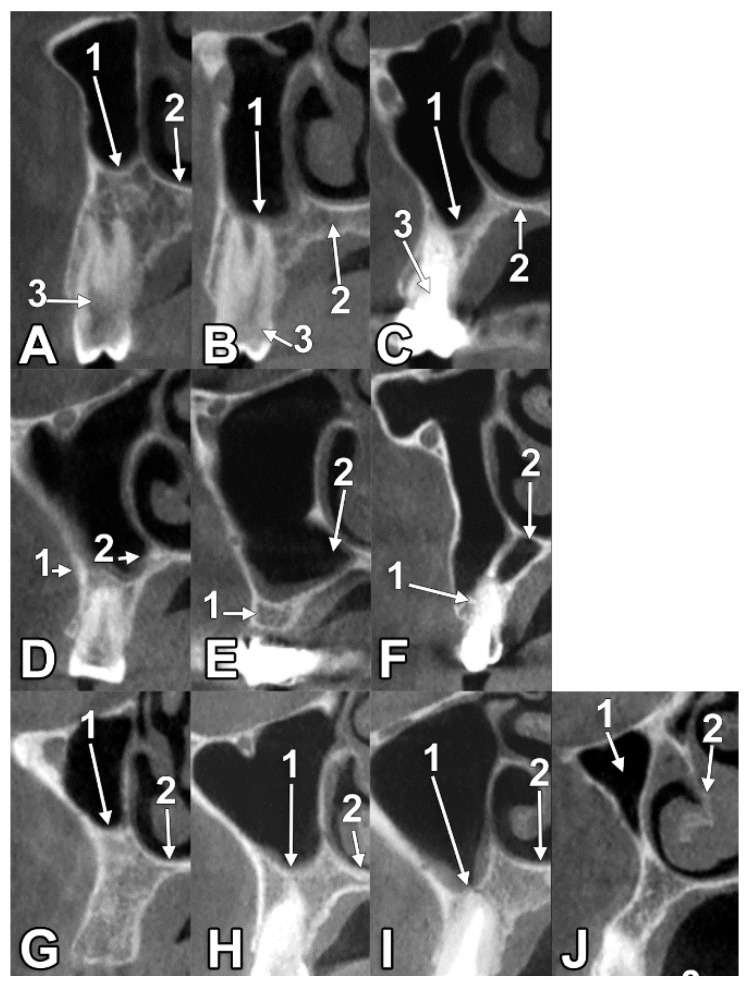
(**A**). Type 1a alveolar base at the right maxillary second premolar level. Orthoradial CBCT slice. 1. Antral alveolar base; 2. nasal floor; 3. second premolar. (**B**). Type 1b alveolar base at the left maxillary second premolar level. Orthoradial CBCT slice. 1. Antral floor; 2. palatal process of maxilla; 3. second premolar. (**C**). Type 1c alveolar base at the right maxillary second premolar level. Orthoradial CBCT slice. 1. Alveolar base; 2. nasal floor; 3. second premolar. (**D**). Type 2bO alveolar base in the region of the second left maxillary premolar. Orthoradial CBCT slice. 1. Alveolar base; 2. opened palatal recess. (**E**). Type 2cO alveolar base in the maxillary premolar region, right side. Orthoradial CBCT slice. 1. Alveolar ridge; 2. opened palatal recess. (**F**). Type 2cC alveolar base at the left maxillary second premolar level. Orthoradial CBCT slice. 1. Alveolar bone; 2. closed palatal recess. (**G**). Type 3a alveolar base at the right upper second premolar. Orthoradial CBCT slice. 1. Antral floor; 2. nasal floor. (**H**). Type 3b alveolar base at the right upper second premolar level. Orthoradial CBCT slice. 1. Antral floor; 2. nasal floor. (**I**). Type 3c alveolar base at the level of the left upper second premolar. Orthoradial CBCT slice. 1. Antral floor; 2. nasal floor. (**J**). Type 4 alveolar base at the left maxillary second premolar level. CBCT orthoradial slice. 1. Maxillary sinus; 2. inferior nasal turbinate.

We have documented, bilaterally, the types and variables of the antral floor at the second upper premolar area (Figure 2). On the right side, type 1a (Figure 2A) was found in 8/145 (5.52%) cases, type 1b in 29/145 (20.0%) cases, type 1c (Figure 2C) in 62/145 (42.76%) cases, types 2cO (Figure 2E) and 3a (Figure 2G) in 17/145 (11.72%) cases each, type 3b (Figure 2H) in 6/145 (4.14%) cases, and types 3c and 4 in 3/145 (2.07%) each. The types 2bO and 2cC were not found on the right side. On the left side, type 1a was found in 5/145 (3.45%) cases, type 1b (Figure 2B) in 35/145 (24.14%) cases, type 1c in 56/145 (38.62%) cases, types 2bO (Figure 2D) and 2cC (Figure 2F) in 1/145 cases (0.69%) each, type 2cO in 20/145 (13.79%), type 3a in 14/145 (9.66%), type 3b in 4/145 (2.76%) cases, type 3c (Figure 2I) in 3/145 (2.07%), and type 4 (Figure 2J) in 6/145 (4.14%) cases. In the total batch of 290 unilateral determinations, type 1a was found in 4.48% of the cases, type 1b in 22.07% of the cases, type 1c in 40.69% of the cases, types 2bO and 2cC in only 0.34% of the cases each, type 2cO in 12.76% of the cases, type 3a in 10.69% of the cases, type 3b in 3.45% of the cases, type 3c in 2.07% of the cases, and type 4 in 3.10% of the cases. The summary of the type 1–4 prevalence by side (N = 145) is presented in Table 1. Thus, type 1 was detected in 195/290 sinuses (67.24%). On the right side, type 1 was identified in 68.28%. On the right side, type 1 was identified in 66.21%.

### 3.2. Alveolar Base Morphometry in Types 2 and 3

We did not record the 2bO and 2cC types on the right side. In the type 2cO cases on the right side (17 cases), the orthoradial width of the alveolar bone averaged 7.16 mm (SD: 3.72 mm), the rectilinear width of the antral plane averaged 8.21 mm (SD: 1.38 mm), and the depth of the palatal recess averaged 4.08 mm (SD: 2.31 mm). In the type 3a cases on the right side (17 cases), the orthoradial width of the alveolar bone averaged 8.03 mm (SD: 1.64 mm), the rectilinear width of the antral plane averaged 4.55 mm (SD: 1.4 mm), and the rectilinear width of the alveolonasal plane averaged 7.83 mm (SD: 2.2 mm). In the type 3b cases on the right side (six cases), the orthoradial width of the alveolar bone averaged 8.55 mm (SD: 2.26 mm), the rectilinear width of the antral plane averaged 7.03 mm (SD: 3.02 mm), and the rectilinear width of the alveolonasal plane averaged 4.78 mm (SD: 0.59 mm). In the type 3c cases on the right side (three cases), the orthoradial width of the alveolar bone averaged 9.66 mm (SD: 1.26 mm), the rectilinear width of the antral plane averaged 9.74 mm (SD: 0.94 mm), and the rectilinear width of the alveolonasal plane averaged 6.18 mm (SD: 1.19 mm) (Table 2).

On the left side, we recorded all the subtypes of types 2 and 3. In the type 2bO case (one case), the orthoradial width of the alveolar bone averaged 9.65 mm, the rectilinear width of the antral plane averaged 10.2, and the open palatal recess depth averaged 2.25 mm. In the type 2cC case (one case), the orthoradial width of the alveolar bone was 8.5 mm, the rectilinear width of the left antral plane was 8.5 mm, and the depth of the palatal recess was 6.33 mm. Type 2cO was present on the left in 20 cases. The orthoradial width of the alveolar bone was 7.73 mm (SD: 4.1 mm), the rectilinear width of the antral plane was 9.07 mm (SD: 1.9 mm), and the depth of the palatal recess was 3.87 mm (SD: 1.97 mm). On the left side, type 3a was identified in 14 cases. The orthoradial width of the alveolar bone averaged 8.45 mm (SD: 1.24 mm), the rectilinear width of the antral plane averaged 4.9 mm (SD: 1.66 mm), and the rectilinear width of the alveolonasal plane averaged 7.35 mm (SD: 2.36 mm). In the type 3b cases on the left side (four cases), the orthoradial width of the alveolar bone averaged 6.95 mm (SD: 0.92 mm), the rectilinear width of the antral plane averaged 5.9 mm (SD: 1.39 mm), and the rectilinear width of the alveolonasal plane averaged 4.93 mm (SD: 1.54 mm). In the type 3c cases on the left side (three cases), the orthoradial width of the alveolar bone averaged 9.5 mm (SD: 1.09 mm), the rectilinear width of the antral plane averaged 8.83 mm (SD: 2.89 mm), and the rectilinear width of the alveolonasal plane averaged 4.86 mm (SD: 1.84 mm) (Table 2).

We found that types 1 and 2, respectively, exhibited a strong level of statistically significant bilateral symmetry (Pearson’s Chi2 = 86.42, *p* < 0.001). The type 3 cases were correlated equally with contralateral types 1 and type 3. The bilateral symmetry for types 1–3 was stronger in the males (Pearson’s Chi2 = 47.83, *p* < 0.001) than in the females (Pearson’s Chi2 = 56.96, *p* < 0.001). There were no statistically significant associations between the sex and the unilateral anatomical type. 

The ANOVA test indicated no statistically significant differences in the alveolar base width on the right side of this determination. However, there were statistically significant differences (larger variations in values) for the width on the right side for both the antral floor (F = 17.26, *p* < 0.001) and the alveolonasal plane (F = 9.82, *p* < 0.001). There were also statistically significant differences on the left side for the antral floor (F = 9.72, *p* < 0.001) and the alveolonasal plane (F = 5.08, *p* = 0.001).

The correlation tests showed a negative linear association (Pearson’s Chi2 = −0.453, *p* = 0.002) between the dimensions of the antral plane and the alveolonasal plane on the right side. Similarly, we also found a negative association between the two variables on the left side (Pearson’s = −0.499, *p* < 0.001). We also found a bilateral positive association between the size of the antral floor (Pearson’s Chi2 = +0.753, *p* < 0.001), reinforcing the bilateral symmetry of its morphology. The bilateral symmetry of the alveolonasal plane was supported by a positive association for that variable (Pearson’s Chi2 = +0.503, *p* = 0.014). 

## 4. Discussion

Different studies have found that there are no significant differences between the right and left MS volume [13,14]; however, the MS volume in males is significantly higher than that in females [13,15], and the MS volume decreases as age increases [13]. Interestingly, such studies did not find or report any anatomical modification of the typical geometry of the MS, such as by sinus recesses [13,14,15,16,17]. When determinations of the antral floor were performed, different variables of dentosinusal topography are determined. Still, the lateral “gliding” of the nasal fossa above the MAB in the second maxillary premolar has not been reported or studied [10,17,18,19,20]. It was determined that the mean height of the adult maxillary sinus floor correlates negatively with the MS volume and, in adults, it is not significantly influenced by the dentition status and rises in proportion to the decrease in the interzygomatic buttress distance, and in proportion to body height and weight [21]. A study on both dentate and edentulous posterior maxillae concluded that following tooth loss in the posterior maxilla, vertical bone height is primarily lost due to the resorption of the alveolar crest and not due to the pneumatisation of the MS [22]. 

In 321 randomly selected maxillary CBCT scans, the MS alone was located above the second premolar in 46.9% of the cases [23]. In our study, the MS was located exclusively above the MAB on 234/290 sides as types 1 and 2, with an overall prevalence of 80.68%.

In the types 1 and 2 that we determined here, both the buccal (lateral) and transalveolar/transcrestal (inferior) approaches of the antral floor for membrane lifting and implant placement [24,25] could be used as they reach the antral floor. Using the transcrestal approaches, the osteotomes may be initially directed palatally and then redirected more vertically to create the final osteotomy for implant placement [26]. However, if the antral–palatal wall is thin, the initial osteotome path must be changed to engage a thicker zone of the MAB [26].

In the type 2 cases, we determined the incidence of the palatal recesses of the MS, which was 11.72% on the right side and 15.17% on the left side, and we correlated it with the vertical topography of the antral floor—higher than the nasal floor, flush with the nasal floor, or lower than the nasal floor. Other studies have focused on such palatal recesses of the MS [27,28,29]. Different authors have regarded the palatal recesses as palatonasal recesses [25,27,28,29,30] (Table 3). The antral–palatal wall of the antral floor was regarded as the inferior angle side of the palatal recess; in such cases, the opening angle of the palatal recess was determined as the angle of the palatal–antral wall and the sinonasal (medial) wall of the MS [25,29]. The angle of the palatal junction was depicted as the angle between the palatal cortical plates of the antral–palatal wall of the sinus floor and that of the palatal process of the maxillary bone [27,28]. In one of the available studies on the palatonasal recess of the MS, the authors found 1315 pneumatisations of different types, including a null type of the palatine process, but, according to their statements, in just 188 cases (376 MSs) [28]; this raises severe doubts on the truthfulness of their results. The same authors indicate that they evaluated the “maxillary sinus palatal process” “gasification” [28]. However, from an anatomical point of view, from the maxillary bone and not the MS project several processes, including the palatal one. Seemingly, closed palatal recesses of the MS have not been found or described previously, which is explained by the rare occurrence of this type (0.69%). Neither the bilateral symmetry nor asymmetry of the palatal recesses were determined when the palatonasal recesses were found and measured [27]. We demonstrate with our results that the palatal recesses are not necessarily bilateral. The incidence we found for palatal recesses is about half of that found previously by Niu et al. (2018). They investigated many cases and found that at the second premolar and first molar sites, 62% of the patients were without recess types of the MS, and 38% exhibited palatonasal recess types [25]. Palatal, or palatonasal, recesses of the MS have been sparsely reported in the literature, and dental practitioners should be aware of such a condition as it could easily be mistaken for pathology [31]. 

The palatal sinus lifting approach via the antral–palatal wall of the MS allows for higher postoperative comfort, less postoperative oedema, less marginal bone loss around implants, and a higher bone density around implants postoperatively [32]. This approach may be used in cases with a deep palatal (palatonasal) recess of the MS, a thick buccal wall of the antral floor, heavy buccal vestibule scarring, a prominent artery within the buccal wall that would be intercepted in the buccal bony window of osteotomy, and also for re-entry augmentation [33,34,35]. The palatal approach of the antral floor is a safe and effective method to complete a sinus augmentation if a buccal approach is impractical [36]. Nevertheless, caution should be exercised when using such palatal approaches to avoid damage to the greater palatine neurovascular bundle [37].

The preoperative CBCT evaluation of patients helps the surgeons decide whether the palatal or the buccal sinus lifting approach should be used [32]. Moreover, a preoperative scan can inform the surgeons if there are anatomical conditions, such as in types 3 and 4 in the present study, to misdirect the palatal approach to the nasal fossa instead of the MS. Jadach et al. (2024) observed that, to date, there is no systematic anatomical classification available that could help clinicians in choosing between the buccal and palatal approach in sinus lift procedures [34]. These authors documented 200 MSs and determined different classes of subantral residual bone height by comparing the thickness of the buccal and palatal antral walls [34]. 

In types 1 and 2, the medial wall of the antral floor is a well-defined antral–palatal wall and was detected here in 80.69% of the investigated MSs. In 16.21% of the sinuses, however, we found a type 3 alveolar base in which the antral–palatal wall became a composite antral–palatal and nasal–palatal wall by widening the nasal floor. In type 4 (3.10%), this wall is exclusively a nasal–palatal one. Therefore, in the palatal approach of the MS floor for sinus lift, these details should be known, cases should be anatomically assessed preoperatively, and the erroneous approach of the nasal floor by this route can thus be avoided for patients with types 3 or 4 of MABs. 

In the highly resorbed maxilla, marked resorption of the lateral alveolar wall may occur [38]. A sinus membrane lift in this situation exposes a narrow antrum, and the mid-alveolar placement of implants will result in penetration directly into the NF [38]. This risk increases in type 3 MABs. If implants penetrate the NF, they could be easily accessed through a small osteotomy of the antral–palatal wall that can later be covered with a membrane [38].

In types 3 and 4, the MAB contributed to a nasal–palatal wall instead of an antral–palatal one on 25/290 sides of our batch, which was equivalent to 8.62%. The nasal–palatal bone could be used for implant placement, and if it is severely resorbed, a nasal lift technique could be considered to augment the available bone. The nasal lift technique combines turbinectomy and lifting the anteroposterior nasal floor through a lateral window using autogenous bone or bone substitutes to augment an atrophied alveolar ridge in the anterior maxilla [39]. 

Inferior nasal meatus pneumatisation increases the extent of the nasal fossa and results in the displacement of the MS laterally and the nasal fossa inferiorly to the posterior maxillary teeth; this was described as the “big-nose variant” [23]. It was shown to have an incidence of approximately 3% [40] and was also reported as a rare case [41]. We found a higher incidence here, of 8.62%. 

A previous study determined that several implants appeared to penetrate the sinus floor on panoramic radiographs. Still, in CBCT, it was demonstrated that a part of these penetrated the nasal floor because the inferior meatus pneumatisation extended up to the second molar area [42]. Indeed, inferior meatus pneumatisation could determine either a type 3 or a type 4 of MAB and should be carefully checked with CBCT and not on panoramic radiographs. Specifically, when the MS appears small, considering that the nasal fossa occupies a broader area in that region, it can be used for an accurate interpretation [43]. 

The sample size of the present study could be regarded as a limitation of it. However, we consider that specific surgical approaches should be decided on a case-by-case basis after documenting the MAB type on CBCT. Artificial intelligence (AI) could be of help for implant planning, but more extensive studies are required to train the AI model for bone measurements [12]. Fontenele et al. (2023) aimed to develop and assess the performance of a novel AI-driven convolutional neural network (CNN)-based tool for automated three-dimensional maxillary alveolar bone segmentation on CBCT images [44]. They reached the conclusion that although the manual segmentation shows slightly better performance, the novel CNN-based tool also provides a highly accurate segmentation of the maxillary alveolar bone and its crestal contour, consuming 116 times less than the manual approach [44].

## 5. Conclusions

In conclusion, the MAB in the upper second premolar area should not be considered exclusively antral when using surgical approaches to the MS floor or in specific anatomical teaching for dentistry. The present study justifies, on one hand, a preoperative CBCT evaluation of the MAB and, on the other hand, the need to perform similar studies in the molar region of the antral floor. 

## Figures and Tables

**Table 1:** The prevalence of types 1–4 (No., %).

Type of Sinus Floor	Right Side	Left Side
1	99, 68.28%	96, 66.21%
2	11, 11.72%	22, 15.17%
3	26, 17.93%	21, 14.48%
4	3, 2.07%	6, 4.14%

**Table 2:** Mean values of the morphometric variables in types 2 and 3. R: right side; L: left side; N: number of cases; SD: standard deviation.

Side and Type		Orthoradial Width of the Alveolar Bone	Rectilinear Width of the Antral Plane	Depth of the Palatal Recess (Type 2) or Rectilinear Width of the Alveolonasal Plane (Type 3)
R 2cO	Mean	7.1600	8.2124	4.0829
	N	17	17	17
	SD	3.72400	1.38610	2.31857
R 3a	Mean	8.0388	4.5535	7.8341
	N	17	17	17
	SD	1.64044	1.40314	2.20125
R 3b	Mean	8.5500	7.0317	4.7800
	N	6	6	6
	SD	2.26288	3.02309	0.59464
R 3c	Mean	9.6600	9.7467	6.1800
	N	3	3	3
	SD	1.26361	0.94638	1.19025
L 2bO	Mean	9.6500	10.2000	2.2500
	N	1	1	1
	SD	-	-	-
L 2cC	Mean	8.5000	8.5000	6.3300
	N	1	1	1
	SD	-	-	-
L 2cO	Mean	7.7325	9.0760	3.8765
	N	20	20	20
	SD	4.10709	1.90907	1.97757
L 3a	Mean	8.4507	4.9000	7.3550
	N	14	14	14
	SD	1.24908	1.66072	2.36808
L 3b	Mean	6.9575	5.9050	4.9375
	N	4	4	4
	SD	0.92579	1.39266	1.54496
L 3c	Mean	9.5700	8.8300	4.8633
	N	3	3	3
	SD	1.09343	2.89876	1.84072

**Table 3:** Previous studies of the palatal/palatonasal recesses of the MS.

Author(s), Year	Method, Lot	Findings Related to the Palatal Recess of the MS
Chan et al., 2013 [29]	CBCT, 225 cases	- The mean PNR location was gradually lower from the 2nd premolar to the 2nd molar sites; - Opening angles of the palatal recesses were determined and were significantly wider in the 2nd molar sites than in the 2nd premolar and 1st molar sites; - In the 2nd premolar sites, the risk of perforation of the Schneiderian membrane was higher (15%) than in the 1st molar sites (8.2%) and 2nd molar sites (2.4%).
Monje et al., 2017	CBCT, 180 cases	- The study was performed in edentulous cases; - A homogenous linear increasing opening angle of the palatal recess was shown for 1st premolar to 2nd molars.
Gunacar et al., 2022 [28]	CBCT, 188 cases	- The presence and depth of the palatal recess, its opening angle, and the angle of the palatal junction were evaluated.
Serindere et al., 2023 [27]	CBCT, 200 cases	- The presence and depth of the palatal recess, its opening angle, and the angle of the palatal junction were evaluated; - Palatal recesses were found in 24.75% of cases.

## Data Availability

The dataset used and analysed during the current study is available from the corresponding author upon reasonable request.

## References

[1] Kilic C., Kamburoglu K., Yuksel S.P., Ozen T. (2010). An Assessment of the Relationship between the Maxillary Sinus Floor and the Maxillary Posterior Teeth Root Tips Using Dental Cone-beam Computerized Tomography. Eur. J. Dent..

[2] Shokri A., Lari S., Yousef F., Hashemi L. (2014). Assessment of the relationship between the maxillary sinus floor and maxillary posterior teeth roots using cone beam computed tomography. J. Contemp. Dent. Pract..

[3] Kang S.H., Kim B.S., Kim Y. (2015). Proximity of Posterior Teeth to the Maxillary Sinus and Buccal Bone Thickness: A Biometric Assessment Using Cone-beam Computed Tomography. J. Endod..

[4] Nimigean V., Nimigean V.R., Maru N., Salavastru D.I., Badita D., Tuculina M.J. (2008). The maxillary sinus floor in the oral implantology. Rom. J. Morphol. Embryol..

[5] Guillou E., Lerhe B., Gemmi T., Khenissa N., Latreche S., Loridon G. (2024). Simultaneous sinus elevation and immediate implant placement without biomaterial: A technical note. J. Stomatol. Oral Maxillofac. Surg..

[6] Zhang L., Zhou C., Jiang J., Chen X., Wang Y., Xu A., He F. (2024). Clinical outcomes and risk factor analysis of dental implants inserted with lateral maxillary sinus floor augmentation: A 3- to 8-year retrospective study. J. Clin. Periodontol..

[7] Mahmood Hashemi H., Mohammadi S., Razmara F. (2024). The Causes of Dental Implant Migration into the Maxillary Sinus: A Case Series Study from 25 Years of Experience. J. Dent..

[8] Ilie A.C., Jianu A.M., Rusu M.C., Muresan A.N. (2022). Anatomical Changes in a Case with Asymmetrical Bilateral Maxillary Sinus Hypoplasia. Medicina.

[9] Hamdy R.M., Abdel-Wahed N. (2014). Three-dimensional linear and volumetric analysis of maxillary sinus pneumatization. J. Adv. Res..

[10] Kwak H.H., Park H.D., Yoon H.R., Kang M.K., Koh K.S., Kim H.J. (2004). Topographic anatomy of the inferior wall of the maxillary sinus in Koreans. Int. J. Oral Maxillofac. Surg..

[11] Hsu Y.T., Rosen P.S., Choksi K., Shih M.C., Ninneman S., Lee C.T. (2022). Complications of sinus floor elevation procedure and management strategies: A systematic review. Clin. Implant Dent. Relat. Res..

[12] Altalhi A.M., Alharbi F.S., Alhodaithy M.A., Almarshedy B.S., Al-Saaib M.Y., Al Jfshar R.M., Aljohani A.S., Alshareef A.H., Muhayya M., Al-Harbi N.H. (2023). The Impact of Artificial Intelligence on Dental Implantology: A Narrative Review. Cureus.

[13] Aktuna Belgin C., Colak M., Adiguzel O., Akkus Z., Orhan K. (2019). Three-dimensional evaluation of maxillary sinus volume in different age and sex groups using CBCT. Eur. Arch. Otorhinolaryngol..

[14] Sarilita E., Lita Y.A., Nugraha H.G., Murniati N., Yusuf H.Y. (2021). Volumetric growth analysis of maxillary sinus using computed tomography scan segmentation: A pilot study of Indonesian population. Anat. Cell Biol..

[15] Sharma S.K., Jehan M., Kumar A. (2014). Measurements of maxillary sinus volume and dimensions by computed tomography scan for gender determination. J. Anat. Soc. India.

[16] Tolstunov L., Thai D., Arellano L. (2012). Implant-guided volumetric analysis of edentulous maxillary bone with cone-beam computerized tomography scan. Maxillary sinus pneumatization classification. J. Oral. Implantol..

[17] Gu Y., Sun C., Wu D., Zhu Q., Leng D., Zhou Y. (2018). Evaluation of the relationship between maxillary posterior teeth and the maxillary sinus floor using cone-beam computed tomography. BMC Oral Health.

[18] Ok E., Gungor E., Colak M., Altunsoy M., Nur B.G., Aglarci O.S. (2014). Evaluation of the relationship between the maxillary posterior teeth and the sinus floor using cone-beam computed tomography. Surg. Radiol. Anat..

[19] Razumova S., Brago A., Howijieh A., Manvelyan A., Barakat H., Baykulova M. (2019). Evaluation of the relationship between the maxillary sinus floor and the root apices of the maxillary posterior teeth using cone-beam computed tomographic scanning. J. Conserv. Dent..

[20] Yoshimine S., Nishihara K., Nozoe E., Yoshimine M., Nakamura N. (2012). Topographic analysis of maxillary premolars and molars and maxillary sinus using cone beam computed tomography. Implant Dent..

[21] Ariji Y., Ariji E., Yoshiura K., Kanda S. (1996). Computed tomographic indices for maxillary sinus size in comparison with the sinus volume. Dentomaxillofac. Radiol..

[22] Schriber M., Bornstein M.M., Suter V.G.A. (2019). Is the pneumatisation of the maxillary sinus following tooth loss a reality? A retrospective analysis using cone beam computed tomography and a customised software program. Clin. Oral Investig..

[23] Haj Yahya B., Bar-Hai D., Samehov D., Chaushu G., Hamzani Y. (2021). Sinus Augmentation-Expect the Unexpected: Diagnostic Anatomical Study. J. Clin. Med..

[24] Albadani M.M., Elayah S.A., Al-Wesabi M.A., Al-Aroomi O.A., Al Qadasy N.E., Saleh H. (2024). A graftless maxillary sinus lifting approach with simultaneous dental implant placement: A prospective clinical study. BMC Oral Health.

[25] Niu L., Wang J., Yu H., Qiu L. (2018). New classification of maxillary sinus contours and its relation to sinus floor elevation surgery. Clin. Implant Dent. Relat. Res..

[26] Bruschi G.B., Bruschi E., Papetti L. (2021). Flapless Localised Management of Sinus Floor (LMSF) for trans-crestal sinus floor augmentation and simultaneous implant placement. A retrospective non-randomized study: 5-year of follow-up. Heliyon.

[27] Serindere G., Serindere M., Gunduz K. (2023). Evaluation of maxillary palatal process pneumatization by cone-beam computed tomography. J. Stomatol. Oral Maxillofac. Surg..

[28] Gunacar D.N., Kose T.E., Arsan B., Aydin E.Z. (2022). Radioanatomic study of maxillary sinus palatal process pneumatization. Oral Radiol.

[29] Chan H.L., Monje A., Suarez F., Benavides E., Wang H.L. (2013). Palatonasal Recess on Medial Wall of the Maxillary Sinus and Clinical Implications for Sinus Augmentation via Lateral Window Approach. J. Periodontol..

[30] Monje A., Urban I.A., Miron R.J., Caballe-Serrano J., Buser D., Wang H.L. (2017). Morphologic Patterns of the Atrophic Posterior Maxilla and Clinical Implications for Bone Regenerative Therapy. Int. J. Periodont. Restorat. Dent..

[31] Omami G. (2013). Palatine recess of maxillary sinus masquerading as radiolucent lesion: Case report. Libyan Dent. J..

[32] EL-prince O.N., Khalil A.F., EL-sabbagh A.M., Fahmy M.H. (2018). Palatal Versus Buccal Antral Approach for Maxillary Sinus Lifting and Implant Placement. Alex. Dent. J..

[33] Rahpeyma A., Khajehahmadi S. (2018). Indications for palatal sinus lift: Case series. J. Indian Soc. Periodontol..

[34] Jadach R., Asa’ad F., Rasperini G., Osypko K. (2024). Classifying Maxillary Sinuses of Polish Patients for Sinus Lift: A Pilot Study. Dent. J..

[35] Seemann R., Wagner F., Ewers R., Ulm C. (2013). Palatal sinus elevation revisited: A technical note. J. Oral Maxillofac. Surg..

[36] Sarmiento H.L., Othman B., Norton M.R., Fiorellini J.P. (2016). A Palatal Approach for a Sinus Augmentation Procedure. Int. J. Periodont. Restorat. Dent..

[37] Ueno D., Kurokawa T., Maruo K., Watanabe T., Jayawardena J.A. (2015). Palatal window osteotomy technique improves maxillary sinus augmentation in previously insufficient augmentation case. Int. J. Implant Dent..

[38] Jensen O.T., Perkins S., Van de Water F.W. (1992). Nasal fossa and maxillary sinus grafting of implants from a palatal approach: Report of a case. J. Oral Maxillofac. Surg..

[39] Camargo I.B., Oliveira D.M., Fernandes A.V., Van Sickels J.E. (2015). The nasal lift technique for augmentation of the maxillary ridge: Technical note. Br. J. Oral Maxillofac. Surg..

[40] Misch C.E., Resnik R. (2017). Misch’s Avoiding Complications in Oral Implantology.

[41] Khoshhal M., Vafaee F., Sabounchi S.S., Sabounchi S.S. (2016). Three-dimensional radiograph revealing the big-nose variant before dental implant surgery in posterior maxilla: A rare case report. Int. J. Med. Res. Prof..

[42] Park W.B., Kim Y.J., Kang K.L., Lim H.C., Han J.Y. (2020). Long-term outcomes of the implants accidentally protruding into nasal cavity extended to posterior maxilla due to inferior meatus pneumatization. Clin. Implant Dent. Relat. Res..

[43] Yeom H.G., Huh K.H., Yi W.J., Heo M.S., Lee S.S., Choi S.C., Kim J.E. (2023). Nasal cavity perforation by implant fixtures: Case series with emphasis on panoramic imaging of nasal cavity extending posteriorly. Head Face Med..

[44] Fontenele R.C., Gerhardt M.D.N., Picoli F.F., Van Gerven A., Nomidis S., Willems H., Freitas D.Q., Jacobs R. (2023). Convolutional neural network-based automated maxillary alveolar bone segmentation on cone-beam computed tomography images. Clin. Oral Implant. Res..

